# P026: Genetic characterization of NSP4 gene form rotavirus infected Saudi children

**DOI:** 10.1186/2047-2994-2-S1-P26

**Published:** 2013-06-20

**Authors:** M Aly, A Alkhairy, S Aljohani, H Balkhy

**Affiliations:** 1 King Abdullah International Medical Research Center, Saudi Arabia; 2 King Abdulaziz Medical City, Riyadh, Saudi Arabia

## Introduction

Rotavirus gastroenteritis in Saudi infants is tremendous and escalating problem. The non-structural viral protein NSP4 is encoded by the tenth segment of the viral genome. NSP4 is a multifunctional which plays an important role in the rotavirus pathogenesis as viral endotoxin. Genetic variations and the prevalent NSP4 genotype in Saudi Arabia remain unidentified

## Objectives

To characterize the genotype range of NSP4 gene among the prevalent rotavirus genotypes at a tertiary care hospital in Saudi Arabia

## Methods

Patient stool samples of children aged 6 years or less admitted to the hospital at King Abdul Aziz Medical City (KAMC) Riyadh with acute diarrhea were collected from inpatients. Samples were identified for rotavirus positive using the ELISA. RNA extractions were done by RNA isolation kit (Magnapure), followed by RT-PCR. NSP4 genes were identified using PCR and sequencing technique to detect the prevalent genotype. NCBI blastn vr2.2 and RotaC^2^ genotyping tools were used to explore the genotypic variation among the positive viral infected children.

## Results

To date, 428 pediatric patients were screened for rotavirus infection between January 2011 and February2012. Preliminary results showed that 39.9% (n=171) faecal samples were positive for HRA. More than 81% were infants less than 2 years, 60.2% (n=103) males and females were 39.7%. There was no significant seasonal effect observed, however, positive samples peaked in July (n=35). G1P[8] is the most prevalent rotavirus genotype in our region with (62%). NSP4 genotype E1 was prevalent in more than 77 % (n= 132) of the positive rotavirus cases.

## Conclusion

Here we identified the **NSP4 E1** genotype as ubiquitous in rotavirus infected Children. Further work is needed to identify the other NSP4 genotypes and explore their genetic diversity among the Saudi infected population.

## Disclosure of interest

None declared

